# The Effectiveness of Adipose Tissue-Derived Mesenchymal Stem Cells Mixed with Platelet-Rich Plasma in the Healing of Inflammatory Bowel Anastomoses: A Pre-Clinical Study in Rats

**DOI:** 10.3390/jpm14010121

**Published:** 2024-01-22

**Authors:** Georgios Geropoulos, Kyriakos Psarras, Maria Papaioannou, Vasileios Geropoulos, Argyri Niti, Christina Nikolaidou, Georgios Koimtzis, Nikolaos Symeonidis, Efstathios T. Pavlidis, Georgios Koliakos, Theodoros E. Pavlidis, Ioannis Galanis

**Affiliations:** 1 2nd Propaedeutical Department of Surgery, Hippokration Hospital, School of Medicine, Aristotle University of Thessaloniki, 54642 Thessaloniki, Greecedrgxkoimtzis@gmail.com (G.K.); niksym@gmail.com (N.S.); pavlidis.md@gmail.com (E.T.P.); pavlidth@auth.gr (T.E.P.); galanis@auth.gr (I.G.); 2 Laboratory of Biological Chemistry, School of Medicine, Aristotle University of Thessaloniki, 54124 Thessaloniki, Greece; mpapaioannou@auth.gr; 3 Biohellenika Biotechnology Company, 55535 Thessaloniki, Greece; argyrw.niti@gmail.com (A.N.);; 4 Department of Histopathology, Hippokration Hospital, 54642 Thessaloniki, Greece; christinapathologist@gmail.com

**Keywords:** mesenchymal stem cells, platelet-rich plasma, anastomosis, intestinal healing, anastomotic leak, colitis

## Abstract

**Introduction:** Multiple factors have been linked with increased risk of anastomotic leak in bowel surgery, including infections, inflammatory bowel disease, patient comorbidities and poor surgical technique. The aim of this study was to investigate the positive effect, if any, of adipose derived mesenchymal stem cells (MSCs) mixed with platelet-rich plasma (PRP) in the healing of bowel anastomoses, in an inflammatory environment after establishment of experimental colitis. **Materials and Methods:** Thirty-five male Wistar rats were divided into five groups of seven animals: normal controls, colitis controls, PRP, MSCs, and PRP+MSCs. All groups underwent laparotomy, one-cm segmental colectomy and anastomosis in situ. In the colitis group, colectomy was performed at the affected area. Colitis was previously established by transrectal administration of 2,4,6-trinitrobenzene sulfonic acid (TNBS) except for the normal controls. Post-mortem histopathological, tissue hydroxyproline and anastomotic bursting pressure (ABP) assessments were performed. The Mann–Whitney U test was used to assess statistical significance differences between groups. **Results:** No perioperative mortality was noted. Tissue hydroxyproline and ABP were significantly increased in the group of PRP+MSCs compared to colitis controls (*p* = 0.0151 and *p* = 0.0104, respectively). Inflammatory cell infiltration was lower and fibroblast activity higher in PRP+MSCs group, but not statistically significant (*p* > 0.05). Neoangiogenesis (*p* = 0.0073) and anastomotic area epithelialization (*p* = 0.0182) were significantly higher in PRP + MSCs group compared to colitis controls. **Discussion:** The synergistic effect of the PRP and MSCs is apparently responsible for the improved healing markers in bowel anastomoses even on inflammatory bowel. This gives hope for primary anastomoses and stoma saving in many emergency and/or elective circumstances, especially in immunocompromised or malnourished patients, even in cases with inflammation or peritonitis. Clinical studies should follow in order to support the clinical application of PRP+MSCs in gastrointestinal anastomoses.

## 1. Introduction

Despite our extended knowledge of the healing process in the gastrointestinal wall, the anastomotic leak is still a major problem in clinical practice. Even after meticulous patient preparation and optimization of factors like anastomotic technique, patient comorbidities and perioperative care, anastomotic leakage may occur in up to 14% of cases but varies depending on the location of the anastomosis and the participating organs [1,2,3]. For example, leakage rates may be up to 17.5% in pancreaticojejunal, 14.6% in esophagojejunal and up to 17% in ileocecal anastomoses [4,5,6]. In cases where the patient presents any reasons for bad healing (elderly, immunocompromised, malnourished, inflammation, infection, etc.), a bowel anastomosis may lead to a detrimental series of septic complications if a leakage occurs. This is riskier in emergency settings with unprepared bowel or in inflammation sites (inflammatory bowel or peritonitis). To avoid such complications, most surgeons prefer to avoid the anastomosis itself by changing the operation to Hartmann’s procedure, or, if they decide to complete the anastomosis in the first setting, most of them choose to establish a prophylactic stoma or ileostomy. Stomas may sound a safe option for the surgeon; however they are not free of complications, and many patients find it quite frustrating, with negative impact on their quality of life [7].

Adipose tissue is an excellent source of mesenchymal stem cells (MSCs), given its abundance in humans and experimental animals. Adipose tissue-derived MSCs have been proven to provide a significant benefit in wound healing via several mechanisms. MSCs secrete significant levels of growth factors, extracellular matrix and cytokines that modulate the immune function [8,9]. Following tissue damage stimuli, a variety of growth factors, including tumor necrosis factor-α (TNF-α), fibroblast growth factor (FGF), vascular endothelial growth factor (VEGF) and transforming growth factor-β (TGF-β) are released. These growth factors have been repeatedly shown to significantly enhance wound healing [8,10]. At sites of tissue damage, the recruited MSCs express the C-X-C chemokine receptor type 4 (CXCR-4) protein, which twists inflammatory cells to present an enhanced wound healing activity [11]. The extracellular matrix proteins secreted by MSCs assist in the angiogenesis and chemotaxis of inflammatory cells that promote wound healing. MSCs constitute 1% of the adipose tissue cellular population. Additionally, MSCs participate in the wound healing process by differentiating into fibroblasts and epithelial cells, and they substitute tissue damage by tissue regeneration via a series of multiple actions mediated by certain receptors and pathways. Moreover, the growth factors secreted by MSCs stimulate cells adjacent to tissue injury to participate in the wound healing process [12]. It is very important that MSCs do not provoke immune reactions, since they do not express any histocompatibility complex antigens. Interestingly, previous studies have shown that when MSCs are incorporated into other biomaterials like chitosan, enhance wound healing by their ability to differentiate into epithelial cells [13]. Furthermore, exosomes and microvesicles secreted by MSCs induce cell migration and wound matrix remodeling with an increased production of collagen [14]. These characteristics make MSCs a valuable tool to use in multiple clinical scenarios.

Platelet-rich plasma (PRP) is produced from blood via simple centrifugation. Several reports have shown that PRP supports wound healing via its high concentration in various growth factors and cytokines including platelet-derived growth factor (PDGF), TGF-β, FGF, VEGF, hepatocyte growth factor (HGF), insulin-like growth factor (IGF), interleukin-1β, interleukin-6 and interleukin-4 [15,16]. PDGF has been shown to improve epithelialization and tissue regeneration via secretion of several other tissue growth factors [17]. Other studies investigating the combination of PRP and MSCs (PRP+MSCs) have demonstrated that VEGF facilitates MSCs’ angiogenic properties and enhances the wound healing process. Finally, fibroblast mitogenic activity is improved by the presence of IGF, which is secreted by PRP [18].

To date, limited literature exists investigating the effect of MSCs or PRP in gastrointestinal wound healing and especially in bowel anastomoses in colitis settings. A very well-established inflammatory bowel disease model is colitis induced via intrarectal administration of 2,4,6- trinitrobenzene sulfonic acid (TNBS), and a wide variety of studies investigating multiple agents in bowel anastomoses healing have been published since [19]. TNBS facilitates Th1 immunologic response and T cell-mediated immune reaction in the exposed tissues, mimicking inflammatory bowel syndromes. Inflammation impairs intestinal wound healing, and wise surgeons would never dare to perform anastomoses in such cases. Therefore, it would be extremely useful to discover ways to enhance healing in inflammatory bowel tissues. This study aims to investigate the healing effectiveness of the combined administration of PRP and MSCs in anastomoses performed in rats with inflammatory bowel disease.

## 2. Materials and Methods

### 2.1. Experimental Animals and Study Groups

The animal study protocol was approved by the Institutional Review Board (protocol code EL-54-BIOexp-10 and date of approval 11 September 2023). Forty male Wistar–albino rats of the same age and body weight were used. The first five rats were the donors for adipose tissue and blood in order to prepare the adipose-derived MSCs and the PRP samples. Rats were maintained separately in cages at 22 °C ± 2 °C and 40–50% humidity with a 12-h light/dark cycle and were allowed free access to tap water and standard laboratory rat pellets. Thirty-five rats were randomly divided into five groups of seven rats each: Group 1: end-to-end colonic anastomosis without experimental colitis, Group 2: experimental colitis + end-to-end colonic anastomosis, Group 3: experimental colitis +end-to-end colonic anastomosis +PRP injection, Group 4: experimental colitis+end-to-end colonic anastomosis +MSCs injection and Group 5: experimental colitis +end-to-end colonic anastomosis + PRP+MSCs injection. The G*Power statistical software (software (Faul, Erdfelder, Lang, & Buchner, 2007, version 3.1.9.6) was used to calculate the sample size.

### 2.2. PRP Preparation

For the purpose of this study, allogeneic PRP was used. A small amount of peripheral blood, approximately 3 mL, was collected from each one of the donors into sterile tubes with EDTA. The tubes were centrifuged for 15 min at 270× *g*, at room temperature. The supernatant was aspirated, and a second centrifugation was executed at 1000× *g* for 7 min, at room temperature. The platelet-poor plasma was discarded, and the pellet was resuspended in EDTA. The PRP was then transferred into cryogenic tubes and stored at −80 °C in 500 μL aliquots. The final volume of platelet-rich plasma that was injected to each anastomosis was 500 μL divided at four sites in each intestinal stump. This corresponded to a total platelet number of 1 × 10^6^ cells/μL. The purpose of the cryopreservation was the induction of platelet activation and alpha-granule release. Frozen PRP was thawed prior to anastomosis application.

### 2.3. Isolation, Characterization, and Culture of Adipose Tissue MSCs

**Figure 1:**
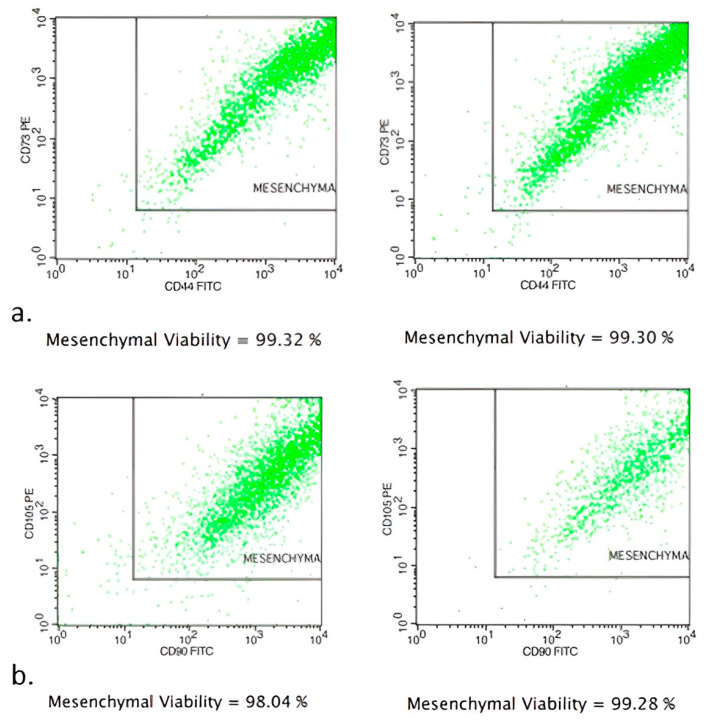
Flow cytometric analysis of MSCs characterized by the expression of MSCs markers. (**a**) MSCs were positive for CD73 (96.19% expression) and CD44 (97.31% expression). (**b**) MSCs were positive for CD90 (96.98% expression) and CD105 (92.46% expression).

**Figure 2:**
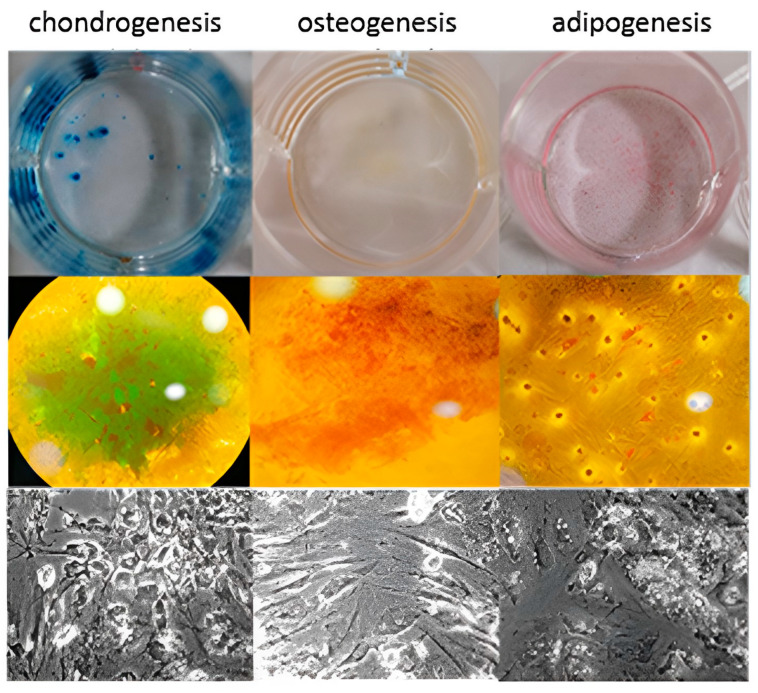
Tri-lineage differentiation (cartilage, bone and adipose tissue) potential of MSCs after 10 days of culture in differentiation mediums. This figure shows that the MSCs used in our study had a significant potential to differentiate and be appropriate for use.

**Figure 3:**
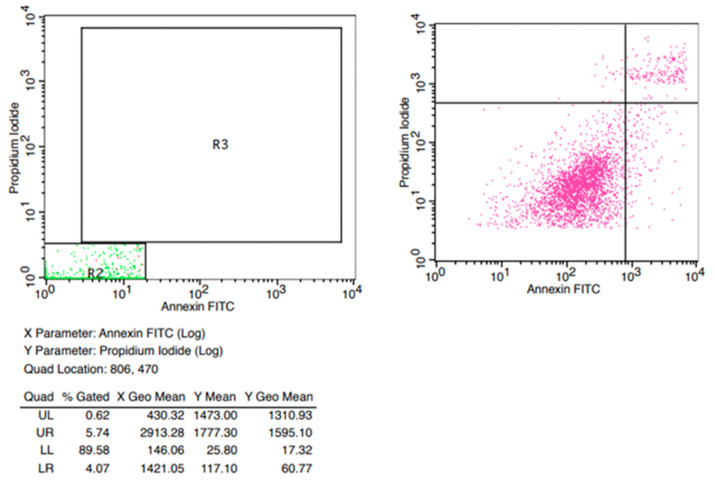
Representative results of flow cytometry for MSCs’ viability after thawing.

The source of adipose tissue and MSCs was allogeneic. Adipose tissue was removed via lipectomy surgeries from the same five donors as aboved and transferred into sterile containers under aseptic conditions. Approximately 2 g of adipose tissue of each sample was cut into fine pieces, washed with normal saline 0.9% to remove red blood cells and digested with 0.075% collagenase type I (Worthington, Columbus, OH, USA) and 1% (*v*/*v*) penicillin/streptomycin (Pen/Strep, Biowest, Nuaillé, France) for 1 h at 37 °C on a shaker. Then, collagenase was removed via dilution with phosphate-buffered saline solution (PBS). The resulting cell suspension was centrifuged at 600× *g* for 20 min. The supernatant was removed, and the pellet was resuspended in low-glucose Dulbecco’s Modified Eagle’s Medium (DMEM, Biowest^®^) supplemented with 10% (*v*/*v*) fetal bovine serum (FBS, Biowest^®^) and 1% (*v*/*v*) Pen/Strep. Isolated cells were multiplied in T150 flasks using low-glucose DMEM 10% (*v*/*v*) FBS and 1% (*v*/*v*) Pen/Strep at 37 °C in a humid atmosphere containing 5% CO_2_ for 7 days. After that, cells were cultured for 7 more days in T75 flasks and finally were stored in cryovials in vapor-phase liquid nitrogen below −150 °C. Flow cytometry analysis showed MSCs to be positive for the expression markers CD90 and CD105. The percentages of positive cell markers and their histograms are given in Figure 1. Moreover, the isolated cells were found to be capable of differentiating into osteocytes, chondrocytes and adipocytes under the appropriate conditions (Figure 1). Differentiation into chondrocytes is shown with production of deposits of acid mucopolysaccharides which were confirmed via Alcian Blue, differentiation into osteoblasts was demonstrated via detection of Alizarin Red S stain-positive calcium deposits, and differentiation into adipocytes was revealed via the formation of cytoplasmic lipid droplets stained with Oil Red O stain (Figure 2). Finally, the stem cells were placed in one-mL microneedle insulin syringes suspended in 500 μL PBS containing 3 × 10^6^ MSCs before their injection into colonic anastomoses circumferentially located at four sites in each intestinal stump. When PRP+MSCs were applied, MSCs were resuspended directly in 500 μL PRP aliquots. Cell viability was checked before injection into the rats. After thawing, a small amount of the cell suspension was used for flow cytometry to determine viability using FITC Annexin V Apoptosis Detection Kit with 7-AAD (EXBIO, Chemical company, Greece)). Fluorescent intensities of the cells were detected using a flow cytometer BD FACSCalibur (BD Biosciences) (Figure 3).

### 2.4. Experimental Colitis

**Figure 4:**
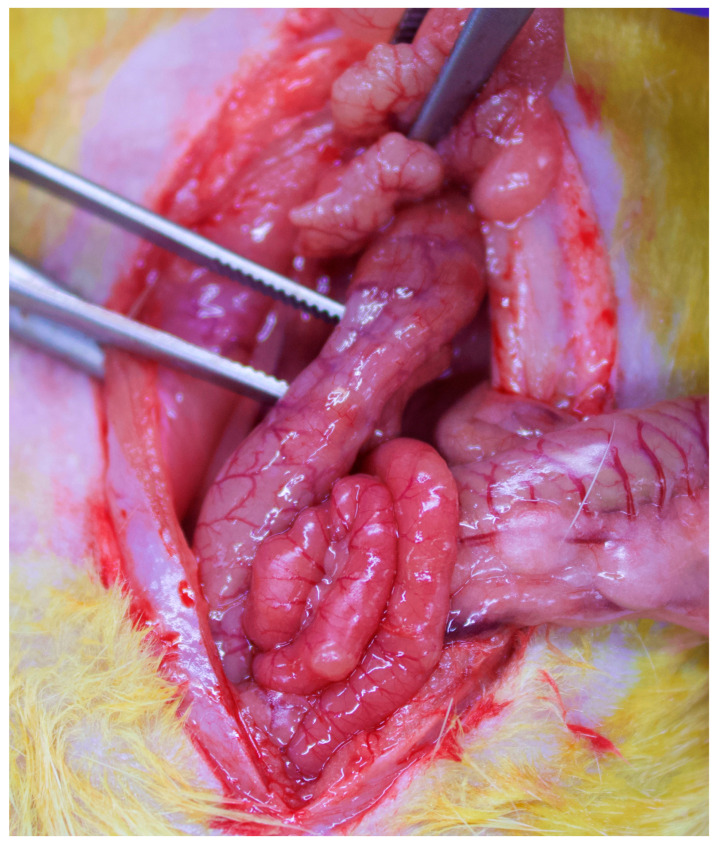
Intraoperative findings of macroscopic colitis. Forceps indicate a large bowel segment that presents signs of acute inflammation: hyperemia, induration, loose adhesions to the surrounding organs and edema.

Experimental colitis was induced via transrectal administration of TNBS (Sigma-Aldrich, St. Louis, MO, USA). A solution of 5 mg TNBS dissolved in 0.5 mL 40% ethanol was prepared to be administered to all animals of groups 2 to 5. Under light ether anesthesia, a PE-50 cannula (8 cm long) was inserted into the colon via the anus, and a 0.25 mL of the above solution (40% ethanol containing 5 mg TNBS) was instilled into the lumen of the colon. Solution diffusion to the proximal colon and prevention of expulsion was performed by placing the animal in reverse position and holding the cannula intrarectally for one minute. All animals were assessed daily for clinical signs of colitis: presence of wet feces in the anal area, loose stools or diarrhea, bleeding per rectum, altered behavior, weight loss and piloerection. This procedure led to similar degrees of colitis. However, in order to avoid bias, the degree of colitis had been previously standardized via primary experiments using several dilutions of TNBS and ethanol and assessing the following parameters upon autopsy (no:0 and yes:1): 10% loss of body weight (0 or 1); wet anus, soft stool, or empty colon (0 or 1); anal bleeding/occult blood (0 or 1); macroscopic ulcers present (0 or 1); and death (0 or 1). To standardize experiments, a score of at least 2 to 3 degrees (score 2 or 3) was decided to be appropriate in order to avoid animal deaths and facilitate comparisons between groups. Also, all included animals should present macroscopic findings of colitis in the surgical specimen (Figure 4). Therefore, all animals having colitis of different degrees at autopsy were discarded and replaced by new ones for the comparisons. In total, 6 animals needed to be replaced for the final comparisons.

### 2.5. Surgical Procedure

Rats were anaesthetized via intraperitoneal injection of ketamine hydrochloride (Ketalar^®^) and xylazine (Rompun^®^) at a dose of 50 mg/kg and 5 mg/kg, respectively. All surgical interventions were performed by the same surgeon (GG). Postoperative analgesia was achieved via intraperitoneal injection of paracetamol at a dose of 500 mg/kg. Following a midline laparotomy, a segmental colectomy 1 cm in length was performed on the inflamed area of the colon at approximately 3 cm’s distance from the anus, as measured via insertion of an intrarectal catheter. The colon was sharply divided by knife, and immediately a 500 μL volume of PRP, MSCs or PRP+MSCs was injected submucosally at a depth of about 1 mm at 4 symmetrical sites in the intestinal wall of both intestinal stumps by making use of a fine (insulin injection) needle. Following that, anastomosis was performed via standard procedures using 8 single sutures of 6-0 Prolene stitches. The abdominal cavity was washed with normal saline and surgically closed. All animals received one dose of ciprofloxacin 500 mg/kg, metronidazole 500 mg/kg and paracetamol 500 mL/kg, intraperitoneally.

### 2.6. Autopsy—Macroscopic Examination

On day seven, all animals were sacrificed via neck dislocation, and the abdominal cavity was reopened and examined by two independent observer surgeons blinded to the interventions. Intraperitoneal adhesions, anastomotic leaks, collections, abscesses, inflammation or damages in surrounding organs were assessed and documented. Anastomotic leak was declared (or suspected) if free bowel content was noted in the abdominal cavity, or any presence of abscess or extensive adhesions in the perianastomotic area. All animals experiencing complications were included in the analysis.

### 2.7. Anastomotic Bursting Pressure (ABP) Measurement

A 4-cm piece of colon containing anastomosis in the middle was connected to a classical sphygmomanometer using a 3-way connector and a drip tube while the other end of the colon was tightly ligated. The colon was dived into a beaker filled with normal saline, and air was pumped into the 3-way entrance of the tube with a slow rate of 5 cc/min via use of a syringe pump. Bursting pressure was recorded at the time that the first air bubbles appeared at the site of anastomosis. Following this, the colon anastomotic site, which was defined as 0.5 cm bilaterally from the anastomotic line, was longitudinally divided into fragments for further evaluations. A portion was fixed in formalin for histology, and another portion was weighted and snap-frozen in liquid nitrogen for collagen measurements.

### 2.8. Histologic Examination

All colon specimens were fixed in formalin and embedded in paraffin. Hematoxylin-eosin stained sections were evaluated by two blinded pathologists without any knowledge of the groups. The specimens were evaluated for fibroblast cell concentration/density, neovascularization, inflammatory cell infiltration and anastomotic area epithelialization. The histopathological results of the groups were assessed according to the Phillips et al. scale [20]. Each of the aforementioned parameters was rated on a scale from 0 to 4: 0: None, 1: Slight increase, 2: Moderate infiltration, 3: Dense infiltration and 4: Confluent cells or fibers. For the assessment of epithelialization, a 0 to 3 scale was used: absent (zero points), incomplete cubic (one point), normal cubic (two points), or normal glandular (three points) [21].

### 2.9. Hydroxyproline Assessment

Tissues were initially weighed and homogenized mechanically via grinding using mortar and pestle and several cycles of freezing/thawing of the tissues in liquid nitrogen. For the assay, high-temperature glass tubes were used. Test samples were analyzed at a concentration of 100 μg/mL, and successive dilutions of a collagen solution (Achille’s tendon, Sigma-Aldrich) were used for the preparation of a standard curve. Test samples/standards were firstly hydrolyzed via autoclaving for 20 min at 120 °C in NaOH (10.125 N). The pH of the samples/standards was subsequently regulated at 6–7 by adding HCl solution (8 N), followed by induction of hydroxyproline oxidation via incubation for 25 min at room temperature in a T-Chloramine solution (0.056 M in 10% isopropanol and 90% acetate-citrate buffer pH 6.5). The chromophore was then developed with the addition of Ehrlich’s solution (1 M) and incubation for 20 min at 65 °C. The absorbance of a reddish complex was measured at 550 nm using a spectrophotometer. The absorbance values were plotted against the concentrations of standards, and the concentration of hydroxyproline in sample tissues was determined from the standard curve in μg/mL.

### 2.10. Statistical Analysis

The G*Power statistical software (Faul, Erdfelder, Lang, & Buchner, 2007, version 3.1.9.6) was used to calculate the sample size. A strong consideration of reduction principle from the 3Rs (replacement, reduction and refinement domains of animals used in research) took place. Data from a previously published animal study was used. An effect size of 0.69, five groups of animals, power of the test 80% and a significance level of *p* < 0.05 indicated a total number of 35 animals [22,23]. The histopathological values were expressed with median, first and third quadrantile. The anastomotic burst pressure and tissue hydroxyproline levels were presented with mean and standard deviations. Overall comparisons between groups for each of the histopathologic domain (inflammatory cell infiltration, angiogenesis, fibroblast activity and anastomotic area epithelialization), tissue hydroxyproline and anastomotic bursting pressure was performed with the Kruskal–Wallis test. When statistically significant differences were found, an unpaired, two-sided Mann–Whitney U test was performed to assess which of the individual groups presented statistically significant differences. A value of *p* < 0.05 was considered to be statistically significant.

## 3. Results

### 3.1. Autopsy

All animals presented clinical findings of colitis that included diarrhea, piloerection and reduced mobility. No mortality was noted throughout the study. Immediate or delayed immunoreactions reactions were not observed. No collections, free fluid or abscesses were detected. A few postoperative complications were detected: abdominal wound dehiscence (1/35 in MSCs group), anastomotic leak (2/35 in colitis control and MSCs group) and ileus (1/35 in colitis control group). The anastomotic leak was suspected to be due to dense adhesions around the anastomosis in two animals as mentioned above. In all other animals, anastomoses were intact without any dense adhesions.

### 3.2. APB

**Figure 5:**
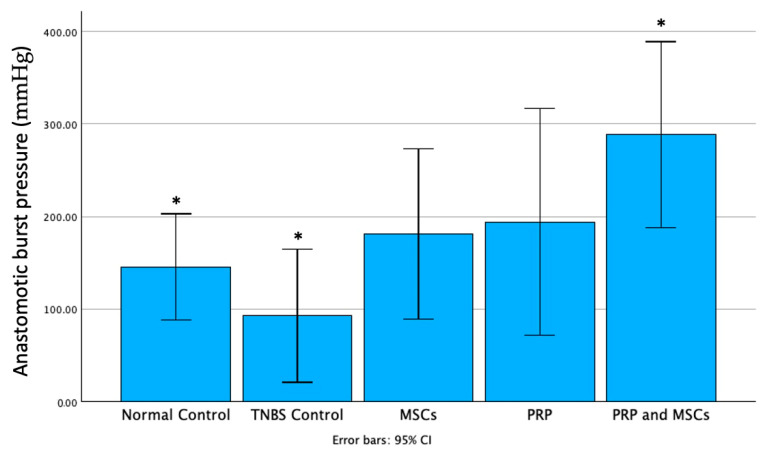
ABP calculation. PRP+MSCs present a statistically significant increased ABP over the TNBS control. Differences between normal control MSCs, PRP and combination of PRP and MSCs are not significant. Data presented as mean ± SD with the 95% confidence interval. Asterisk (*) indicates statistically significant differences.

All anastomoses were subjected to APB measurement. In two cases, dense adhesions were observed around the anastomotic site. These two specimens were transferred into the ABP measuring devise en bloc, along with their adhesions. This was to keep the anastomosis intact while measuring the ABP. These samples exhibited very low ABP compared to the mean (65 and 80 mmHg), obviously due to leakage. All other anastomoses did not present any suspicion of perforation. The mean ± SD values of the anastomotic burst pressures were: 145.71 ± 62.14 (Group 1), 92.85 ± 77.82 (Group 2), 181.42 ± 99.57 (Group 3), 194.28 ± 132.39 (Group 4) and 288.57 ± 108.69 (Group 5) (Table 1). Animals treated with PRP+MSCs presented significantly higher ABPs compared to the normal (*p* = 0.0477) and colitis (*p* = 0.0104) control groups. The APB in the remaining group combinations did not reveal any statistical difference (Figure 5).

### 3.3. Histopathological Examination

**Figure 6:**
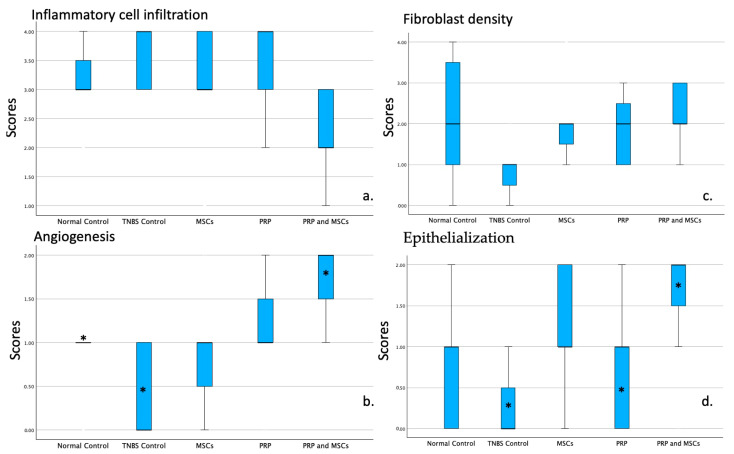
Histopathology box plots of the inflammatory cell infiltration (**a**), angiogenesis (**b**), fibroblast density (**c**) and anastomotic area epithelialization (**d**). Each of the aforementioned parameters scored on a scale from 0 to 4: 0: None, 1: Slight increase, 2: Moderate infiltration, 3: Dense infiltration and 4: Confluent cells or fibers. For the assessment of epithelialization a 0 to 3 scale was used: absent (zero points), incomplete cubic (one point), normal cubic (two points), or normal glandular (three points). Values were presented as median, first, third quartile and 95% confidence interval. Asterisk (*) indicates statistically significant differences.

**Figure 7:**
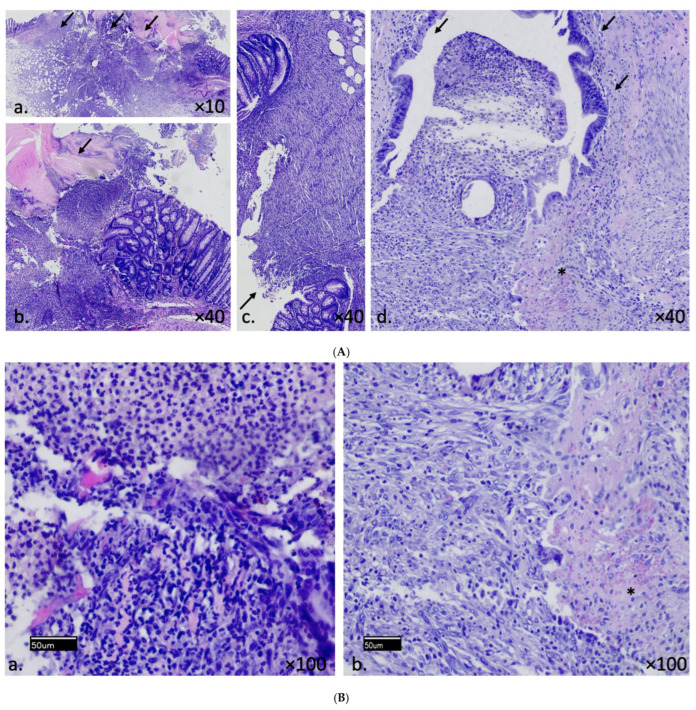
(**A**) Histopathological appearance of colonic anastomotic site in colitis control group (**a**,**b**) and PRP+MSCs treatment group (**c**,**d**). Arrows indicate the anastomotic line area. Colitis control group shows increased anastomotic area distance and absence of epithelialization (**a**), also friable anastomotic area tissue with solely inflammatory cell infiltration without evidence of fibrous tissue formation (**b**). In PRP+MSCs group there is prominent collagen deposition along with inflammatory cell infiltration (**c**), evidence of muscular tissue formation (*) and progenitor epithelial cells starts to cover the anastomotic area along with underlying dense capillaries ((**d**), arrows). (**B**) Greater magnitude (×100) of the colitis control and PRP+MSCs group at the anastomotic line area. (**a**) Colitis control group: dense inflammatory cell infiltration with up to 30% necrosis. (**b**) PRP+MSCs group: less inflammatory cell deposition, evidence of neoangiogenesis with visible small vessel structures (*) with up to 5% necrosis.

The inflammatory cell infiltration, angiogenesis, fibroblast density and anastomotic area epithelialization were assessed. Specifically, regarding the inflammatory cell infiltration, despite being lower in the PRP+MSCs group, no statistically significant difference was found when compared to the rest of the groups (*p* = 0.0832). Angiogenesis was significantly higher in PRP+MSCs group compared to the TNBS colitis (*p* = 0.0073) and normal control (*p* = 0.0214) group. Epithelialization scores were significantly higher in the PRP alone (*p* = 0.348) and PRP+MSCs (*p* = 0.0182) group compared to the colitis control. Fibroblast activity was higher in the PRP group alone and PRP+MSCs group; however, no statistically significant differences were found in all group comparisons (*p* = 0.1118). Table 1 summarizes the median (first quartile, third quartile) of the histopathological scores as well as the tissue hydroxyproline and ABP mean ± standard deviation values. The plots in Figure 6 show the differences between controls and PRP, MSCs and PRP+MSCs groups. Histopathological photos presenting the improved healing of PRP+MSCs compared to the colitis control group is shown in Figure 7A,B.

### 3.4. Hydroxyproline Levels

**Figure 8:**
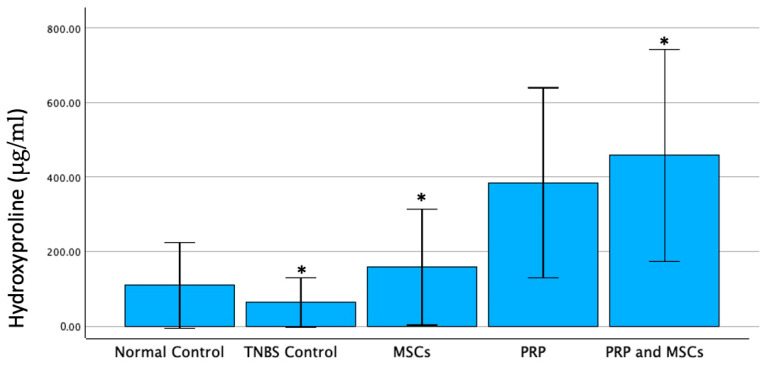
Tissue hydroxyproline levels. Tissue hydroxyproline was significantly higher in the MSCs group (*p* = 0.03) and PRP+MSCs group (*p* = 0.0151) compared to the TNBS colitis control group. Tissue hydroxyproline in the remaining group combinations did not reveal any statistical difference. Asterisk (*) indicates statistically significant differences.

Hydroxyproline levels reflect collagen production in healing tissues. The mean ± SD values for the tissue hydroxyproline levels were as follows: 109.14 ± 125.05 (Group 1), 63.3 ± 72.29 (Group 2), 158.38 ± 168.17 (Group 3), 387.71 ± 275.55 (Group 4), 457.95 ± 307.08 (Group 5). Our results revealed a statistically significant difference between groups. More specifically, tissue hydroxyproline was significantly higher in the MSCs (*p* = 0.03) and PRP+MSCs (*p* = 0.0151) groups compared to the TNBS colitis control group. Tissue hydroxyproline in the remaining group combinations did not reveal any statistical difference. Therefore, there is collagen upregulation in the MSCs and much more in the PRP+MSCs group (Figure 8).

## 4. Discussion

To the best of our knowledge, this is the first study investigating the effect of the combined administration of PRP and adipose tissue-derived MSCs on inflammatory bowel anastomoses after the establishment of experimental colitis in rats. Our results indicate that the infiltration of the enteric stumps with a combination of these two agents gives an impressive boost in the enteric wall healing even on inflammatory bowel. This result may be due to enhanced collagen deposition, as indicated via the significantly increased tissue hydroxyproline levels in the PRP+MSCs group samples. Additionally, the significantly increased neovascularization and epithelialization at the anastomotic line of PRP+MSCs group may also have contributed to the intestinal healing improvement. PRP alone or MSCs alone showed improved anastomotic area healing compared to colitis controls, however, that was not statistically significant; therefore, these two agents need to be applied together in order to exert their synergistic effect. Apparently, the PRP environment, which is rich in nutrients and growth factors, may act as a medium for MSCs to efficiently heal the anastomotic site. Furthermore, reduced inflammatory cell infiltration was detected; thus, PRP+MSCs may also mediate certain anti-inflammatory actions, as has been previously described by others [8,9].

The healing process seems to differ in different parts of the gastrointestinal tract like the stomach and small and large bowel [24,25]. Previous studies have shown that colonic anastomosis has a slower rate of healing compared to the ones performed in the ileum. Small bowel anastomosis reaches its normal strength in about 4 weeks, in contrast to large bowel anastomosis, which, even after 4 months, gains only 75% of its strength [26,27]. This fact also potentiates the need to find agents that could enhance the colonic anastomotic healing, especially in pathologic conditions like ischemia or inflammation, where the healing process is impaired. The healing process of bowel anastomosis is a series of overlapping events. Three classic stages have been described: the exudative, the proliferative and the remodeling phase [6]. Systemic inflammatory cells like neutrophils, macrophages, platelets and fibroblasts exert their role at the exudative phase. Platelets are responsible for the cessation of further blood loss from the bowel wound edge, but not exclusively. Platelets secrete multiple growth factors as well. These factors induce cell migration, proliferation, extracellular matrix synthesis and angiogenesis. Other factors that promote angiogenesis include hypoxia, EGF, endothelin 1, TNF-a, interleukin 1b, monocyte chemotactic protein-1 (MCP-1) and macrophage inflammatory protein (MIP-1a) [28]. After two to fourteen days, the proliferative phase is characterized by cellular proliferation, angiogenesis and reproduction of extracellular matrix. At this stage, macrophages that reside at the anastomotic area serve as growth factor-secreting cells, which promote the formation of the epithelium. In addition, on the fourth postoperative day, fibroblasts are the most prominent cells at the anastomotic site. The presence of PDGF, TGF-b, FGF and IGF-I mostly regulate fibroblast activity. It is quite clear that both MSCs and PRP, with their derivative factors, participate actively in all stages of the intestinal wound healing process [29]. Our study results indicate that PRP+MSCs led to reduced inflammatory cell infiltration, although non-significant. The improved healing in of the PRP+MSCs group may have been assisted by reduced inflammatory cell infiltration. Previous studies have shown that MSCs may reduce inflammatory cell infiltration in tissues and may facilitate the healing process [30,31].

Application of MSCs or PRP have been separately tested in bowel anastomoses in previously experimental animal studies. Administration of stem cells in the anastomotic area in animal models with ischemia or radiation injury report a lower occurrence of anastomotic leaks [32]. Alvarenga et al. reported that adipose tissue-derived MSCs in a TNBS colitis rat model significantly reduce certain tissue inflammatory cells (CD4+ T cells, macrophages) and certain tissue inflammatory cytokines (IL-17, TNF-a and IFN-γ) [33]. A systematic review on the effect of PRP in bowel anastomosis included 16 animal studies. Their results indicate that PRP is safe to administer and provides a significant benefit in animals with peritonitis or post-intraperitoneal chemotherapy application. However, compared with normal controls, PRP did not provide any significant benefit over the bowel anastomosis healing process [18]. These findings are keeping in line with our results where the PRP+MSCs group provided a significant benefit in bowel anastomosis healing over the colitis control group, however when compared with normal controls, no significant changes were observed. Another example of clinical application of MSCs include the Crohn’s colitis perianal fistulas. An early clinical trial in 2005 showed that adipose-derived MSCs showed an up to 60% healing rate of perianal fistulas [34]. Panes et al., in their randomized clinical trial of Darvadstrocel (Alofisel^®^), one of the first commercially available adult MSCs suspension, reported its effectiveness and safety of use for treating complex perianal Crohn’s fistulas [35]. Considering the facts (firstly, that MSCs are commercially available; secondly, that PRP is easily produced via a simple venipuncture/centrifugation and thirdly, the outcomes of our study), future clinical studies of PRP+MSCs in humans could be seriously considered.

Co-administration of MSCs and PRP has also been investigated in several experimental and clinical settings of other pathologies. Ebrahim et al. showed that their combined administration significantly improved epithelialization, collagen deposition and angiogenesis in wound healing of diabetic foot ulcers [36]. In a preclinical model of osteoarthritis, there was improved healing of the articular cartilage with MSCs preconditioned in PRP. Combination of MSCs and PRP seems to augment collagen and proteoglycan contents in the knee joint [37]. A clinical study by Gentile et al. concluded that when adipose tissue-derived MSCs were mixed with PRP for breast fillings, the overlying post-radiotherapy damaged skin was improved [38]. These findings are consistent with the results of our study, since the application of PRP+MSCs led to improved epithelialization and neovascularization at the anastomotic site.

Collagen formation is possibly the main factor leading to stronger bowel anastomoses. It has been reported that the submucosa layer of the bowel wall plays a significant role in supporting the tensile strength of bowel anastomoses. In the submucosa layer the collagen type I predominates, followed by types III and IV. Type I and III collagens are the predominant ones in the granulation and scar tissue following bowel anastomosis [39]. The significant increase in the tissue hydroxyproline levels suggest a considerable increase on collagen production at the anastomotic site which was further higher in the PRP+MSCs group rather than in the MSCs alone group.

Neovascularization is another critical factor for efficient healing. Our study shows that the PRP+MSCs increases vascular formation at the anastomotic area. Surgeons know very well that impaired blood supply to enteric stumps leads to healing failure and anastomotic disruption, therefore they always care to leave intact as much vasculature as possible during dissection. Several studies exist to support the value of neovascularization, which seems to be upregulated by MSCs. Chen et al. showed that MSCs preconditioned with PRP exhibited improved neovascularization in ischemic rat hind limb models [40].

In the clinical setting, gastrointestinal anastomoses are an everyday routine for the general surgeon, and traditionally, there are certain technical rules for good results without complications, which are basic for any surgery trainee. The stumps must be well mobilized to permit free peristalsis, should not be inflammatory (if there is any site of inflammation, it should be excised), well vascularized to permit free blood supply, and finally good and meticulous technique in suturing the layers of the gastrointestinal tract should be followed. This should also be accompanied by efficient parenteral nutritive support, use of drains and patience of both the patient and the surgeon. Nevertheless, whatever measures are taken, often anastomoses are disrupted, and that can be sometimes disastrous, especially at sites where direct vision and manipulations are not easy, and the use of staplers is the only solution, such as in the esophageal or rectal stumps; surgeons can never be sure of the technical efficiency of anastomoses. In fact, the percentage of anastomotic leaks is as high as 17.5% in pancreaticojejunal anastomoses, 14.6% in esophagojejunal anastomoses and up to 17% in ileocecal anastomoses [4,5,6]. These numbers are even higher in immunocompromised or malnourished patients and even more so in emergency and infective/inflammatory settings. Consequently, the results of such complications are very often detrimental, leading to mediastinitis or peritonitis, severe sepsis, systemic inflammatory response syndrome, reoperations, extra enterectomies, new anastomoses and vice versa [41].

Therefore, finding an agent that improves intestinal wound healing and allows for the safe performance of bowel anastomoses, thus avoiding a stoma, was one of the targets of this study. To avoid risky anastomoses, surgeons have the option of reverting stomas whenever applicable. Colostomies and ileostomies have been the gold standard in aid of anastomoses. Stomas have saved lives of many patients, especially in emergency settings. Hartmann’s procedure is the most frequent operation on the colon in all departments of surgery around the globe for obstructive ileus of the left colon, either neoplastic or inflammatory. Transverse colostomies and reverting ileostomies are performed in similar pathologies of the transverse and right colon respectively. Prophylactic stomas, created centrally, are another solution for those surgeons who dare to perform anastomoses in risky cases. However, except the surgeon, not all others are happy with the stoma solution. Patients have difficulty in accepting them, their families often have difficulty supporting patients, and nursing staff are often unhappy in approaching patients. The total cost of hospital stay, re-admissions, patient training, reoperations, disposable materials for stomas etc. seems to be tremendous [42].

Therefore, in surgical practice whenever any inflammatory environment, such as peritonitis, colitis, enteritis, etc., is present, anastomoses should be avoided. Stoma creation, either permanent or prophylactic, is absolutely necessary. On the other hand, and very unfortunately, inflammatory bowel diseases such as ulcerative colitis and Crohn’s disease have been constantly increasing over the past decades in the Western world [43]. Similarly, and even more so, diverticulitis and its complications have been dramatically increasing even in the young population due to extensive stress, fast food remedies and lack of fiber in modern Western dietary habits [44]. Moreover, ischemic colitis is also increasing [45]. Very lately, pseudomembranous colitis has seemed to start a new chapter of severe inflammatory and life-threatening septic situations due to antibiotic overuse [46]. Finally, post-radiation colitis is also a new problem due to therapeutic cancer remedies in the pelvis [47]. Although stomas are a safe classical solution in such cases, in the new era of surgery, a more sophisticated way should be applied in order to strengthen gastrointestinal anastomoses. MSC and PRP co-administration seem to give hope for such a target, as the results of our study suggest. This can be further explained. On the cellular level, multipotent progenitor cells seem to somehow recognize the needs of the tissue where they are placed; they possibly mediate local inflammation decrease via cytokine regulation, and they transform into fibroblasts to produce the correct collagen fibers, while they promote neovascularization and epithelialization of the tissue to make it appropriate [48]. The exact mediators for such a behavior need to be further investigated.

In our experiments, we used heterogenic adipose tissue-derived MSCs. Whether this category of stem cells is appropriate for application in anastomoses is not clear. Nevertheless, it is a feasible approach that can be easily generalized, and it is safe as well. Neither did we observe any significantly increased complications such as stenoses, ileus, immunoreactions or rejection. Besides, as previously commented, heterologous adipose tissue MSCs have already been commercialized in vials and used for the treatment of Crohn’s fistulas in patients, with encouraging results [49]. If they work well for fistulas why not work well for anastomoses either, they could easily be mixed with PRP prepared from the same patient and be applied even in the emergency settings. Additionally, autologous stem cells from the patient could also be prepared if there is at least 1 month time before any scheduled operation [50]. Recently, a rapid adipose tissue MSCs isolation method (isolation of stem cells within 8 h after fat removal) has been described [51]. Is there a need for that or not, that also remains to be investigated. Possibly, heterologous stem cells are safe enough for this purpose, and we do not need to try further. A clinical study incorporating many of these questions is in need of approval by our institution, and hopefully it will soon be scheduled in multiple settings. In our experiments, we simply injected the mixture of MSCs and PRP into the intestinal wall with fine needle insulin syringes at four sites of the intestinal stump periphery. This is the simplest way to do this; however, we can think of more efficient forms of administration. For example, absorbing material for sutures, or biodegradable circular membranes infused with the active solution, or even manufacturing staplers to deliver the active solution along with stapling would be excellent ideas to make surgery safe and effective for our purpose for a strong intestinal anastomosis.

## 5. Conclusions

In conclusion, our study supports the use of MSCs+PRP in inflammatory bowel anastomoses, with excellent results in strengthening anastomotic healing with increased collagen deposition, neovascularization and improved epithelialization at the anastomotic site. The translation of this study to the clinical practice requires further research in clinical and pre-clinical settings to establish the safety and effectiveness of MSC and PRP co-administration in gastrointestinal anastomoses in humans.

## Figures and Tables

**Table 1:** Summary of the histopathological results. Numbers for the histopathological markers are presented in median (first quartile, third quartile) and for ABP (anastomotic bursting pressure) and tissue hydroxyproline in mean ± SD. Asterisk (*) indicates statistically significant differences.

Group	Inflammatory Cell Infiltration	Angiogenesis	Fibroblast Density	Epithelialization	ABP (mmHg)	Tissue Hydroxyproline (μg/mL)
Normal Control	3 (3, 4)	1 (1, 1) *	2 (1, 4)	1 (0, 1)	145.71 ± 62.14 *	109.14 ± 125.05
Colitis Control	4 (3, 4)	1 (1, 1) *	1 (0, 1)	0 (0, 1) *	92.85 ± 77.82 *	63.3 ± 72.29 *
MSCs	3 (3, 4)	1 (0, 1)	2 (1, 2)	1 (1, 2) *	181.42 ± 99.57	158.38 ± 168.17
PRP	4 (3, 4)	1 (0, 1)	2 (1, 3)	1 (0, 1)	194.28 ± 132.39	387.71 ± 275.55 *
PRP and MSCs	2 (2, 3)	2 (1, 2) *	2 (2, 3)	2 (1, 2) *	288.57 ± 108.69 *	457.95 ± 307.08 *

## Data Availability

The data presented in this study are available on request from the corresponding author (accurately indicate status).
